# Plasma Leptin Is Elevated in Acute Exacerbation of Idiopathic Pulmonary Fibrosis

**DOI:** 10.1155/2016/6940480

**Published:** 2016-08-25

**Authors:** Mengshu Cao, Jeffery J. Swigris, Xin Wang, Min Cao, Yuying Qiu, Mei Huang, Yonglong Xiao, Hourong Cai

**Affiliations:** ^1^Department of Respiratory Medicine, Nanjing Drum Tower Hospital, The Affiliated Nanjing University Medical School, Nanjing, Jiangsu 210008, China; ^2^Interstitial Lung Disease Program, Department of Medicine, National Jewish Health, University of Colorado Denver, Denver, CO 80206, USA

## Abstract

*Background*. The natural history of idiopathic pulmonary fibrosis (IPF) is very complex and unpredictable. Some patients will experience acute exacerbation (AE) and fatal outcomes.* Methods*. The study included 30 AE-IPF patients, 32 stable IPF (S-IPF) patients, and 12 healthy controls. We measured the plasma concentrations of leptin and KL-6. Simple correlation was used to assess associations between leptin and other variables. Plasma leptin levels were compared between AE-IPF and S-IPF subjects, decedents, and survivors. Kaplan-Meier curves were used to display survival and Cox proportional hazards regression was used to examine risk factors for survival.* Results*. In subjects with AE-IPF, plasma leptin was significantly greater than in subjects with S-IPF (*p* = 0.0003) or healthy controls (*p* < 0.0001). Plasma leptin was correlated with BMI, KL-6, LDH, CRP, and PaO_2_/FiO_2_ (*p* = 0.007; *p* = 0.005; *p* = 0.003; *p* = 0.033; and *p* = 0.032, resp.). Plasma leptin was significantly greater in 33 decedents than in the 23 survivors (*p* = 0.007). Multivariate Cox regression analysis showed leptin (>13.79 ng/mL) was an independent predictor of survival (*p* = 0.004).* Conclusions*. Leptin could be a promising plasma biomarker of AE-IPF occurrence and predictor of survival in IPF patients.

## 1. Introduction

The natural history of idiopathic pulmonary fibrosis (IPF) is characterized by decreasing pulmonary function over time. Some patients may experience acute exacerbations (AE)—sudden worsening of pulmonary function, increased oxygen requirements, new opacities on chest imaging, and a diffuse alveolar damage (DAD) pattern of lung injury without an identifiable etiology [1]. Estimates vary widely, but each year, a significant minority of IPF patients experience an AE (AE-IPF); about 50% of these patients require ICU admission, and 80% die within 30 days [2–4]. A circulating biomarker is needed to allow clinicians to more precisely evaluate disease activity, in particular, to identify patients at risk for developing AE-IPF and to predict outcomes in patients who suffer AE. Serum carbohydrate antigen-6 (Krebs von den Lungen-6 or KL-6), lactate hydrogenase (LDH), and surfactant proteins A and D (SP-A and SP-D) levels have been evaluated as biomarkers of IPF; each possesses some predictive ability, but none is perfect [2, 5–8].

Leptin was first discovered and cloned by Zhang et al. in 1994 [9]. It is a versatile 16 kDa peptide hormone product of the Obese (OB) gene, and its main function is to regulate energy balance [9, 10]. Leptin is primarily produced by adipocytes in proportion to the size of fat stores. Recently, studies have demonstrated that multiple tissues and cells can produce and secrete leptin, such as liver stellate cells, placenta, gastric fundic mucosa, pancreas, and lung tissue [10–13]. The leptin receptor (LR) exists on hemopoietic precursors, immune cells, vascular endothelium, liver, adipose, and lung tissues [10–14]. Beyond its metabolic function in energy regulation, leptin is implicated in various other physiological processes, including the immune response, inflammatory reactions, and the development of carcinomas, cardiovascular, nervous system, chronic liver, and several respiratory diseases [12–15], including obstructive sleep apnoea (OSA), obesity hypoventilation syndrome (OHS), chronic obstructive pulmonary disease (COPD), and acute lung injury (ALI) [13, 16–18]. To our knowledge, there are no published studies on leptin in IPF or AE-IPF.

In addition to leptin's potential role in ALI, evidence suggests it has a critical role in fibrogenesis programs in the liver, kidney, and lung [14, 19, 20]. For example, leptin-resistant mice are protected from bleomycin-induced pulmonary fibrosis [18]. In normal human lung fibroblasts, leptin augments the transcription of profibrotic genes induced by transforming growth factor-beta 1 (TGF-*β*1) [18]. Among patients with ALI or acute respiratory distress syndrome (ARDS), levels of leptin in bronchoalveolar lavage (BAL) fluid correlate with TGF-*β*1 [18]. Given these data, we speculate that leptin maybe an important factor in the development of AEs and a biomarker for AE-IPF.

The purpose of this study was to measure the expression of leptin in peripheral blood of subjects with AE-IPF and S-IPF. We aimed to begin to build the case for leptin as a biomarker of AE-IPF occurrence and severity by comparing plasma leptin levels in patients with AE-IPF versus those with stable IPF (S-IPF) and by examining correlations between plasma leptin and clinical variables among IPF patients.

## 2. Materials and Methods

### 2.1. Study Population

The study sample consisted of 62 patients with IPF (30 with AE-IPF and 32 with S-IPF) and 12 healthy controls evaluated at Nanjing Drum Tower Hospital, Nanjing, University Medical School from October 2009 to September 2014. The diagnoses of IPF and AE-IPF were made in accordance with published criteria [1, 2]. Stable IPF (S-IPF) means the clinical symptoms, pulmonary function tests, and chest imaging are stable at least one month before collecting peripheral blood. Clinical data were obtained from medical records on admission and follow-up. The study was approved by the Ethics Committee at Nanjing Drum Tower Hospital and conducted in accordance with the principles set forth under the Declaration of Helsinki (1989). All subjects have been consent to participate by signing the informed consent paperwork.

### 2.2. Measurement of Plasma Leptin and KL-6

Peripheral blood samples were collected in evacuated tubes containing ethylene diamine tetraacetic acid (EDTA) from AE-IPF, S-IPF patients and healthy controls in the early morning on empty stomachs. The date of blood collection for patients was the day following hospital admission (including stable IPF patients who were admitted for the diagnoses or following up). The plasma and cells were isolated by centrifugation. The plasma concentrations of leptin (Millipore Corporation, USA) were measured by Enzyme Linked Immunosorbent Assay (ELISA) according to the manufacturer's protocols. KL-6 plasma levels (FUJIREBIO INC, Japan) were assayed by Chemiluminescent Enzyme Immunoassay according to the manufacturer's instructions. All samples were analyzed in duplicate.

### 2.3. Collection of Clinical Data

Clinical data were abstracted from medical records. Vital status was ascertained from medical records or follow-up telephone calls on November 24, 2014. Survival time was calculated from the date of blood collection to the date of death or vital status ascertainment.

### 2.4. Statistical Analysis

Data are presented as mean ± standard deviation (SD) or as counts as appropriate. Differences in values for continuous variables between AE-IPF, S-IPF, and healthy controls were compared by using the Kruskal-Wallis test. The correlations between plasma levels of leptin and clinical variables were analyzed by Spearman correlations. Survival curves were generated for each group using Kaplan-Meier estimates and compared by using the log-rank test. The optimal cut-off value for predicting mortality was decided by ROC curve. A multivariate* Cox* regression model was built to examine leptin as a predictor of time-to-death while controlling for age and oxygenation. With only 30 deaths, including more than three predictors would lead to model over fit; thus, we selected clinically relevant variables to control while examining the effects of leptin on the outcome. ROC curves were used to assess the performance of leptin as a marker of AE-IPF (versus S-IPF) or death. We considered *p* < 0.05 to represent statistical significance. Statistical analyses were performed using IBM SPSS version 19 (SPSS, Inc., Chicago IL, USA) and Prism version 5 (GraphPad, San Diego, CA, USA).

## 3. Results

### 3.1. Clinical Characteristics of Patients with AE-IPF and S-IPF

Baseline characteristics of 62 subjects with IPF are presented in Table 1. The mean age of the 12 healthy controls (males/females: 10/2) was 59.50 ± 4.89 years.

### 3.2. Plasma Level of Leptin Was Elevated in AE-IPF Patients

**Figure 1:**
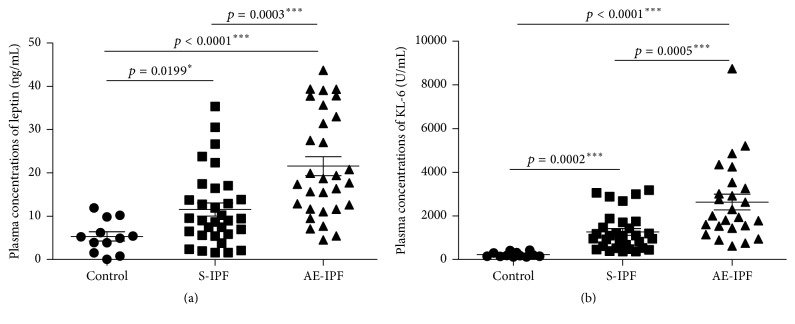
Plasma leptin and KL-6 concentrations in normal controls, S-IPF, and AE-IPF patients. (a) Plasma leptin concentrations were elevated significantly in AE-IPF patients when compared with S-IPF patients and healthy controls by ELISA (*p* = 0.0003 and *p* < 0.0001, resp.). Plasma leptin in S-IPF patients was also increased significantly compared with normal controls (*p* = 0.0199). (b) Plasma KL-6 levels were enhanced significantly in AE-IPF patients compared with S-IPF patients and healthy controls (*p* = 0.0005 and *p* < 0.0001, resp.). Plasma KL-6 in S-IPF patients was also significantly higher than that in normal controls (*p* = 0.0002). ^*∗*^ *p* < 0.05, ^*∗∗∗*^ *p* < 0.001.

In subjects with AE-IPF, leptin concentration was significantly greater than in subjects with S-IPF or healthy controls (Figure 1(a)). Results were similar for KL-6 (Figure 1(b)).

### 3.3. Correlation Analysis between Plasma Leptin and Clinical Variables in 62 IPF Subjects

Leptin concentration correlated with several clinical variables (Table 2).

### 3.4. Plasma Leptin Independently Predicts the Mortality in IPF Patients

**Figure 2:**
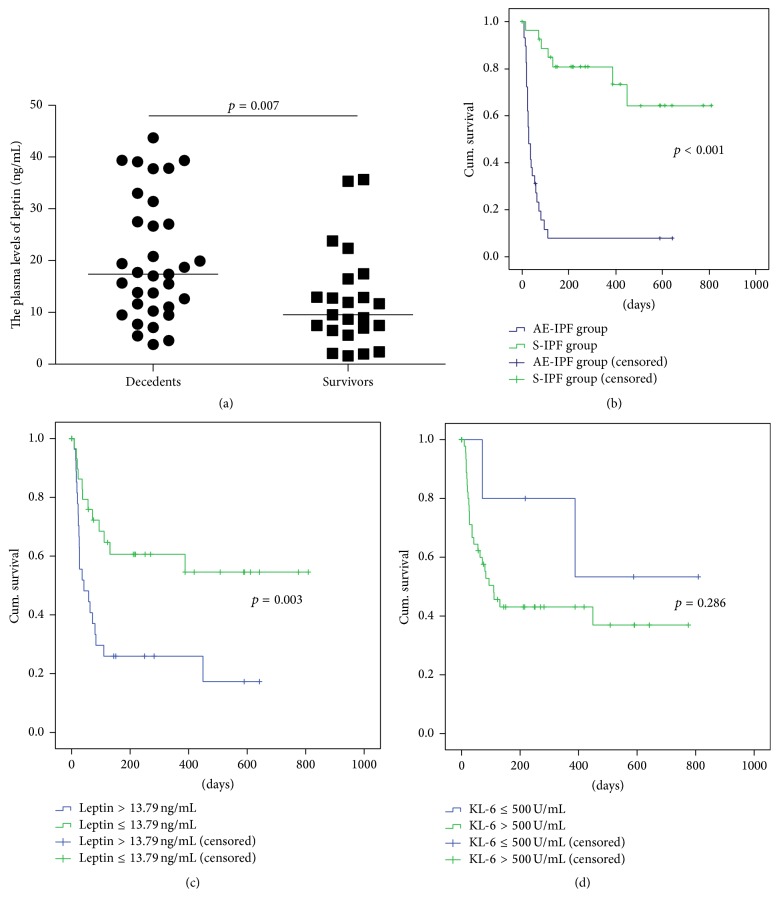
Plasma leptin can predict the mortality of IPF patients independently. (a) Plasma leptin was significantly elevated in decedents (*n* = 33) than that in survivors (*n* = 23) (*p* = 0.007). (b) Kaplan-Meier analyses showed patients with AE-IPF had a significantly higher mortality rate compared with S-IPF subjects by log-rank test (*p* < 0.001). (c) Patients with plasma leptin levels above 13.79 ng/mL had a higher mortality than those with leptin levels lower than that (*p* = 0.003). (d) The survival had no significant difference between patients with plasma KL-6 levels >500 U/mL and those ≤500 U/mL (*p* = 0.286).

Of the 62 subjects with IPF, 33 died, 23 survived, and in 6, vital status was unable to be ascertained. Plasma leptin was significantly higher in decedents than survivors (Figure 2(a)). There was no difference (*p* = 0.71) in plasma leptin between decedents (*n* = 26, 22.41 ± 12.29 ng/mL) and survivors (*n* = 3, 20.08 ± 13.51 ng/mL) in the subgroup of subjects with AE-IPF, nor did plasma leptin discriminate between decedents (*n* = 7) and survivors (*n* = 21) in the S-IPF subgroup (13.57 ± 7.16 versus 10.86 ± 8.43 ng/mL, *p* = 0.203).

Survival of subjects with AE-IPF was significantly worse than subjects with S-IPF (log-rank, *p* < 0.001) (Figure 2(b)). According to the cut-off value for leptin from ROC curve predicting mortality was 13.79 ng/mL. Compared to subjects with leptin levels ≤13.79 ng/mL, those with levels >13.79 ng/mL had shorter survival (log-rank, *p* = 0.003) (Figure 2(c)). For survival among subjects whose KL-6 level was greater than the standard, accepted cut-off value (500 U/mL) was no different from subjects whose KL-6 level was ≤500 U/mL (*p* = 0.286) (Figure 2(d)) [21].

In a multivariable* Cox* model that included leptin and controlled for two other clinically important predictors, leptin was the independent predictor of time-to-death (Table 3).

### 3.5. ROC Curve Analyses for Predicting AE and the Death of IPF Patients according to the Plasma Leptin

**Figure 3:**
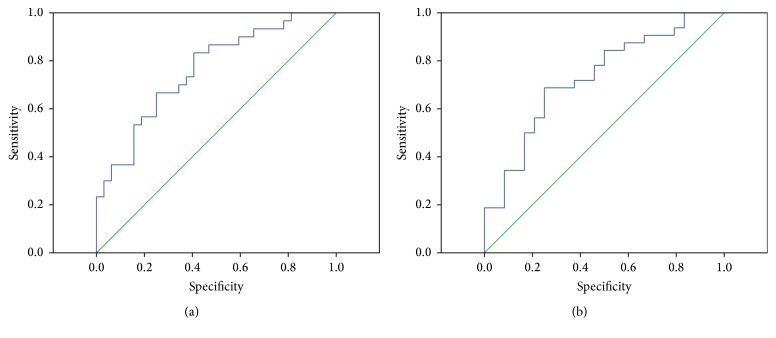
ROC curve analyses for IPF patients according to the plasma levels of leptin. ((a)-(b)) The areas under ROC curves of leptin were statistically significant in the classification of AE-IPF subjects (*n* = 30) or S-IPF patients (*n* = 32) and the decedent (*n* = 33) or the survivor (*n* = 23) (*p* < 0.001, cut-off value 15.52 ng/mL and *p* = 0.004, cut-off value 13.79 ng/mL, resp.).

The cut-off values of plasma leptin predicting AE and death for IPF patients were 15.52 ng/mL (sensitivity 67.86%, specificity 75%) and 13.79 ng/mL (sensitivity 68.75%, specificity 75%), respectively. The area under the ROC curve for leptin in distinguishing AE-IPF from S-IPF was 0.761 (95% CI, 0.644–0.879; *p* < 0.001) (Figure 3(a)) and for distinguishing decedents from survivors was 0.729 (95% CI, 0.596–0.862; *p* = 0.004) (Figure 3(b)).

CRP is also significantly elevated in patients with AE-IPF than S-IPF (shown in Table 1). By comparing the areas under ROC curves, we found plasma leptin (0.761, *p* = 0.000; 0.729, *p* = 0.003) was a better biomarker of IPF acute exacerbation and survival than CRP (0.734, *p* = 0.002; 0.690, *p* = 0.010) (Supplementary Figures 1(A) and 1(B) in Supplementary Material available online at http://dx.doi.org/10.1155/2016/6940480).

## 4. Discussion

In this study, we assessed plasma leptin concentrations in patients with IPF and in healthy controls and found the following: leptin concentration was higher in subjects with AE-IPF than those with S-IPF and higher in subjects who died than in those who survived to vital status ascertainment. Survival was significantly shorter for patients with leptin levels above, compared with those below, 13.79 ng/mL, and leptin was an independent predictor of survival when controlling for age and oxygenation.

The causes and mechanism of acute exacerbation in patients with IPF remain unclear, although it is well known that patients with AE-IPF have significant morbidity and high mortality [1, 4]. Identifying a biomarker to predict IPF disease activity (particularly the occurrence of AE) and outcome of the disease is needed. Elevated serum levels of SP-A, SP-D, KL-6, and CCL18 have been found to be associated with acute exacerbation of IPF [8, 22]. However, these tests are not readily available, and their performance characteristics, while decent, are not perfect. Our results suggest leptin may be a reasonable alternative to these markers.

Leptin is a proinflammatory cytokine and plays an important role in the pathogenesis of ARDS, liver, and lung fibrosis [14, 18, 23]. Peripheral blood leptin is a poor predictor of hepatitis C virus-related fibrosis and represents a negative prognostic factor for response to lamivudine monotherapy in these patients [24, 25]. Furthermore, in patients with ARDS, increased levels of BAL fluid leptin are associated with adverse outcomes [18]. Studies have identified the lung as a leptin-responsive and leptin-producing organ. Several cells in the lungs produce leptin and express OB-Rb [13]. Suggestions that leptin could play a key role in lung fibrosis include that it augments TGF-*β*1 signaling in lung fibroblasts; it does so by inhibiting PPAR*γ*. In addition, leptin signaling is required for bleomycin-induced lung fibrosis [18]. Leptin resistance can protect ALI-susceptible animals from a leptin-mediated inflammatory response to hyperoxia [17]. Additional compelling data have emerged from the COPD literature: during acute exacerbation of COPD, circulating leptin levels increased inappropriately. This is hypothesized as being related to temporary disturbances in the energy balance and the systemic inflammatory response [26, 27]. We suspect leptin may be involved in the development of acute exacerbations of IPF, perhaps by mediating the inflammatory response to injury.

The mortality of AE-IPF subjects was significantly higher than S-IPF subjects, consistent with the published reports [2–4]. In addition, subjects with plasma leptin levels above the cut-off value had significantly shorter survival than those under cut-off value. In contrast, KL-6 was unable to discriminate survivors from decedents in our study, although results from other studies suggest it may be useful for evaluating disease activity and predicting the clinical outcomes [28]. The multivariate analysis showed that even while controlling for other predictors known to possess prognostic value, leptin remained an independent predictor of time-to-death. ROC curves also demonstrated that leptin can differentiate AE-IPF patients from S-IPF patients and predict the survival of IPF patients.

This study has limitations. First, the number of patients and normal controls enrolled was small, and the sample was entirely Asian (Chinese). These may substantially limit the ability to generalize results to the IPF universe, including the 13.79 ng/mL cut-off value. We have no data on the within-subject variability of leptin; levels from the same patient in different clinical circumstances (e.g., before, during, and after AE and when entirely stable) would be informative and provide data to further support the utility of leptin as a biomarker of disease. A prospective, multicenter, and multinational study of a larger patient cohort would be useful to help provide additional data on leptin.

## 5. Conclusions

In summary, we found that leptin was elevated in AE-IPF and that high plasma leptin concentrations are associated with poor survival. Additional research is needed to confirm and extend these results, to determine whether and how leptin plays a role in the pathogenesis of AE, and to delineate the utility of plasma leptin as a biomarker of AE-IPF occurrence and predictor of survival in IPF patients.

## Supplementary Material

Plasma leptin level (0.761, p = 0.000; 0.729, p = 0.003) was a better marker of IPF exacerbation and survival than CRP (0.734, p = 0.002; 0.690, p = 0.010).

## Figures and Tables

**Table 1:** Baseline clinical characteristics of patients with AE-IPF and S-IPF.

Clinical characteristics	*n*	AE-IPF patients	S-IPF patients	*p* value
Gender (male/female)	62	23/7	28/4	0.272
Age (years)	62	64.97 ± 9.36	67.87 ± 8.42	0.206
Smoker (yes/no)	59	9/21	11/18	0.528
BMI (kg/m^2^)	45	24.59 ± 2.71	24.15 ± 2.89	0.679
Leptin (ng/mL)	62	21.60 ± 11.97	11.57 ± 8.54	<0.001^*∗∗∗*^
KL-6 (U/mL)	56	2632.96 ± 1181.50	1264.10 ± 862.51	<0.001^*∗∗∗*^
WBC (×10^9^)	62	10.10 ± 5.42	8.71 ± 3.82	0.244
LDH (U/L)	62	381.07 ± 153.68	241.13 ± 62.84	<0.001^*∗∗∗*^
ESR (mm/h)	62	40.30 ± 27.50	30.38 ± 27.40	0.171
CRP (mg/L)	62	56.74 ± 63.08	20.7 ± 38.27	0.008^*∗∗*^
ALB (g/L)	62	33.97 ± 4.12	37.73 ± 5.07	0.002^*∗∗*^
PaO_2_/FiO_2_	62	204.53 ± 73.22	327.76 ± 66.66	<0.001^*∗∗∗*^
MV (user/nonuser)	62	9/21	1/31	0.004^*∗∗*^
Survival state (dead/censor)	62	26/4	7/25	<0.001^*∗∗∗*^

^*∗∗*^ *p* < 0.01; ^*∗∗∗*^ *p* < 0.001.

**Table 2:** Bivariate correlation analysis between plasma leptin levels and clinical variables among 62 patients with IPF.

Cinical variables	Spearman correlation coefficients	*p* value
BMI (kg/m^2^)	0.418	0.007^*∗∗*^
KL-6 (U/mL)	0.369	0.005^*∗∗*^
WBC (×10^9^)	0.154	0.232
LDH (U/L)	0.367	0.003^*∗∗*^
ESR (mm/h)	0.169	0.201
CRP (mg/L)	0.271	0.033^*∗*^
PaO_2_/FiO_2_	−0.275	0.032^*∗*^

^*∗*^ *p* < 0.05; ^*∗∗*^ *p* < 0.01.

**Table 3:** Cox proportional hazards models for time-to-death among 62 patients with IPF.

Clinical variables	Univariate model			Multivariate model		
	HR	95% CI	*p* value	HR	95% CI	*p* value
Age (>70 yrs old)	0.495	0.247–0.992	0.048	0.647	0.312–1.345	0. 244
Leptin (>13.79 ng/mL)	0.358	0.175–0.734	0.005	0.328	0.153–0.705	0.004
PaO_2_/FiO_2_ (<300)	0.255	0.124–0.524	<0.001	0.256	0.124–0.530	<0.001
