# Supplementary figures and images for: Plasma Leptin Is Elevated in Acute Exacerbation of Idiopathic Pulmonary Fibrosis
Source: Mediators Inflamm. 2016 Aug 25;2016:6940480. doi: 10.1155/2016/6940480 (PMC5014970; doi:10.1155/2016/6940480)

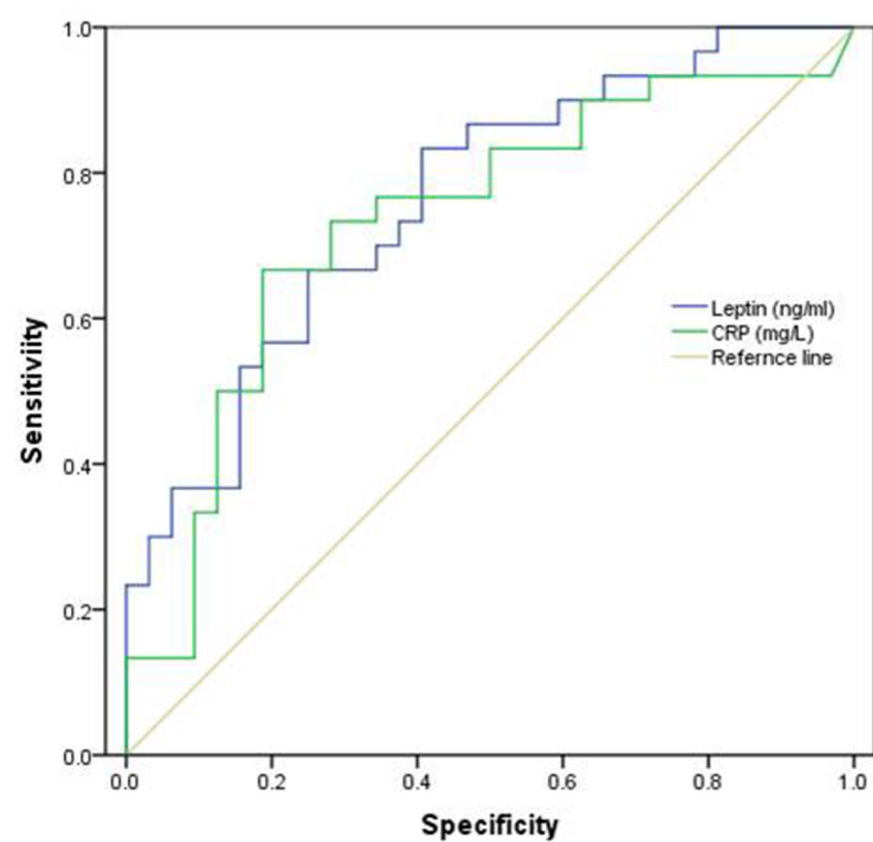

A

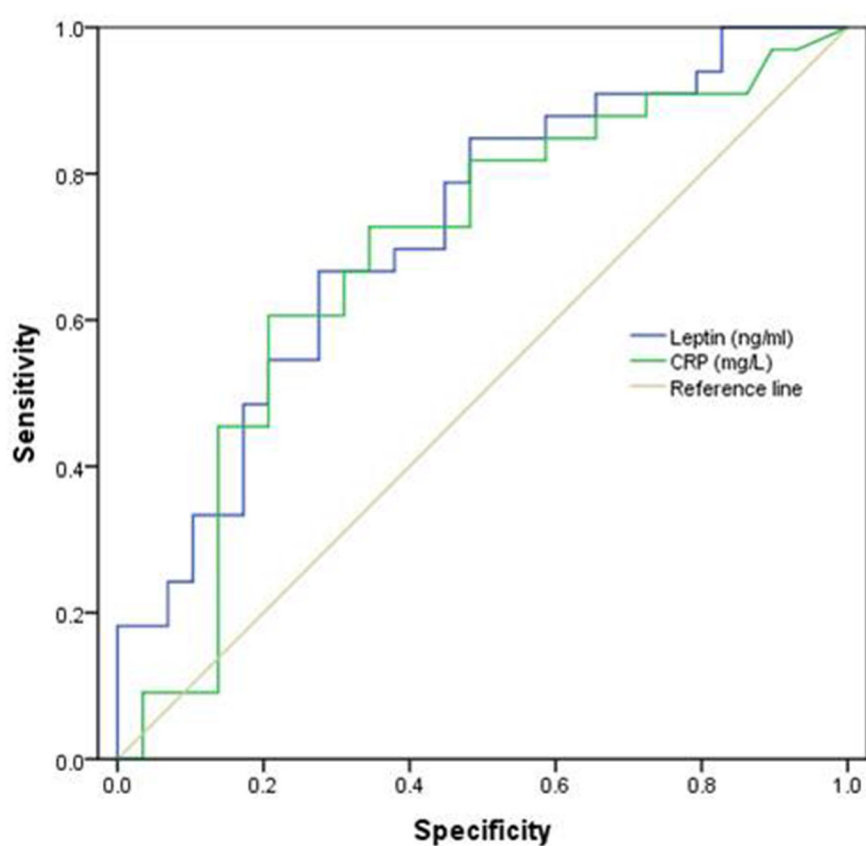

B

Supplement: Supplementary file 1 — Plasma leptin level (0.761, p = 0.000; 0.729, p = 0.003) was a better marker of IPF exacerbation and survival than CRP (0.734, p = 0.002; 0.690, p = 0.010). [file 6940480.f1.pdf]
